# Smoking in Relation to Age in Aesthetic Facial Surgery

**DOI:** 10.1007/s00266-012-9913-2

**Published:** 2012-06-19

**Authors:** An E. K. Deliaert, M. E. P. van den Elzen, E. van den Kerckhove, S. Fieuws, R. R. W. J. van der Hulst

**Affiliations:** 1 Department of Plastic Surgery, Maastricht University Medical Center, P.O. Box 5800, 6202 AZ Maastricht, The Netherlands; 2 Department of Plastic Surgery, Maxima Medical Center, Eindhoven, The Netherlands; 3 Department of Rehabilitation Sciences and Kinesiology, University Hospitals, Leuven, Belgium; 4 Department of Physical Medicine and Rehabilitation and Burns Center, Katholieke Universiteit Leuven, Leuven, Belgium; 5 I-Biostat, Katholieke Universiteit Leuven, Leuven, Belgium; 6 I-Biostat, Universiteit Hasselt, Diepenbeek, Belgium; 7 Department of Plastic Surgery, Viecuri Medical Center, Venlo, The Netherlands; 8 Department of Plastic Surgery, Orbis Medical Center, Sittard, The Netherlands

**Keywords:** Aesthetic surgery, Aging, Dermatochalasis, Smoking, Skin aging, Superior blepharoplasty

## Abstract

**Background:**

Smoking is a major cause of premature facial aging. Skin aging in general, often accompanied by wrinkling and furrowing, plays a significant role in the decision to undergo aesthetic surgery. Smoking may therefore be related to the demand for cosmetic surgery. This study aimed to compare smoking habits with respect to a standard cosmetic procedure (blepharoplasty) in the general population and to evaluate whether the age at surgery differs between smokers and nonsmokers.

**Methods:**

A questionnaire was sent to 517 patients with valid reports describing dermatochalasis of the upper eyelid who subsequently underwent an upper-eyelid correction in 2004. Smoking habits, socioeconomic status, and medical history were evaluated. The patients were classified as smokers, ex-smokers with at least 1 year of smoking cessation, and never-smokers.

**Results:**

Of the 353 questionnaires (68.3 %) returned, 345 were eligible for statistical analysis. The smoking habits did not differ between the blepharoplasty group and the general population. However, the smokers underwent surgery an average of 3.7 years earlier than the ex-smokers (*p* = 0.0007) and 3.5 years earlier than the never-smokers (*p* = 0.006). No significant difference was observed between the ex-smokers and the never-smokers.

**Conclusions:**

This is the first study to describe an association between smoking habits and an earlier need for upper-eyelid correction among ex- and never-smokers. The mechanism of skin restoration could result in a regenerative mechanism among ex-smokers, but further research is needed to support this hypothesis.

**Level of Evidence III:**

This journal requires that authors assign a level of evidence to each article. For a full description of these Evidence-Based Medicine ratings, please refer to the Table of Contents or the online Instructions to Authors at www.springer.com/00266.

## Introduction

Facial aging is characterized by excessive skin, furrows, and wrinkling. Cigarette smoke and chronic sun exposure are thought to be factors contributing to premature skin aging [1–4].

In addition to its deleterious effect on the skin, smoking is particularly known for its carcinogenic effect and the development of cardiovascular and chronic obstructive pulmonary diseases. Moreover, it results in a disturbed microcirculation and subsequent delayed wound healing [5]. Face-lifting is one of the procedures preferably not performed for smokers because of the disturbed vascularization [6, 7]. On the other hand, it is likely that smokers will develop typical skin-aging features earlier in life and may therefore feel the need for cosmetic surgery at an earlier age.

However, a relation between smoking and cosmetic surgery has never been evaluated. Therefore, this study aimed to compare smoking habits between a group undergoing a standard cosmetic procedure (blepharoplasty) and the general population and to determine whether smokers undertake this procedure at an earlier age.

## Materials and Methods

The study was performed between January and December of 2004 at the University Medical Center of Maastricht and at the VieCuri Medical Center Venlo, both situated in the south of the Netherlands. Patients were admitted to the study if a superior blepharoplasty was indicated. Until 2005, this surgical procedure was reimbursed by the patients’ insurance companies. To avoid socioeconomic status bias, we included only patients who underwent surgery in 2004.

The indication to perform an upper-eyelid correction was determined by a plastic surgeon or a senior resident in plastic surgery. For inclusion in the study, the patients had to have valid complaints that were consistent with physical findings indicating that the complaints were amenable to surgical improvement. The complaints were excess skin of the upper eyelid, upper-eyelid folds, and periorbital wrinkles. Moreover, the complaints had to be associated with a subjective decreased range of vision. Contraindications were Graves’ disease and inflammatory disorders of the eyelid.

A questionnaire was sent to all patients (*n* = 517) who underwent an upper-eyelid correction. Informed consent was included in the questionnaire, and written consent was obtained. The questionnaire was subdivided into two sections. In the first section, questions concerning gender, age, and education were asked. The highest-attained education level was defined as lower education (elementary or junior high school), middle education (middle or high school), or higher education (college or university).

The second section focused on medical history and smoking status. The patients were classified as smokers, ex-smokers, or never-smokers. Smokers were asked to report the daily number of cigarettes smoked and their age when they began smoking. Ex-smokers were defined as those who had not smoked for at least 1 year. They were asked to indicate the number of years they had smoked and the year when they quit smoking. In addition, questions were asked about personal care and lifestyle.

The percentages of smokers, ex-smokers, and never-smokers were related to the expected distribution in the Dutch population based on data from the Continuous Survey of Smoking Habits (DCSSH). This cross-sectional population survey of respondents 15 years of age or older is used on a weekly basis to monitor the smoking habits of the Dutch population. The DCSSH is conducted by TNS NIPO, a Dutch Institute for Public Opinion and Market Research [8].

### Statistical Analyses

Kruskal–Wallis, Mann–Whitney *U*, and Fisher’s exact tests were used to make comparisons among smokers, ex-smokers, and never-smokers. Chi-square tests were used to compare the smoking behavior in the current sample with that in the Dutch population, taking sex distribution into account. The percentages were based on subjects for whom the information was complete. All analyses were performed using the statistical package SAS (version 9.1; SAS Institute Inc., Cary, NC, USA). All *p* values lower than 0.05 were considered significant.

## Results

Of the 353 patients (68.3 %) who returned a completed form, eight were excluded due to relocation (*n* = 5), death (*n* = 1), or refusal to participate (*n* = 2). A total of 345 questionnaires were used for statistical analysis. The mean age of all the patients at the time of operation was 54.9 ± 9.3 years. The mean body mass index (BMI) was 25.4 ± 4.6 kg/m^2^.

This study enrolled 61 male and 284 female patients (17.7 vs 82.3%). Information on smoking status was available for 323 patients (93.6%), 86 (26.6%) of whom were smokers who smoked an average of 13.8 ± 7.1 cigarettes a day. Their smoking had begun at a mean age of 20.0 ± 9.6 years. These patients included 135 ex-smokers (41.8%) who had stopped smoking an average of 20.4 ± 11.0 years before the surgical procedure. Also included were 102 never-smokers (31.6%).

Table 1 presents a comparison of data relating to BMI, age, education level, and sun bed use among the smokers, ex-smokers, and never-smokers. The smokers underwent surgery 3.7 years earlier than the ex-smokers (*p* = 0.0007) and 3.5 years earlier than the never-smokers (*p* = 0.006). The ex-smokers and never-smokers did not differ significantly.

**Table 1:** Comparison among smokers, ex-smokers, and never-smokers

	Smokers	Ex-smokers	Never smokers	*p* Value^a^
	(*n* = 86)	(*n* = 135)	(*n* = 102)	
	*n* (%)	*n* (%)	*n* (%)	
Mean age at operation (years)	52.0 ± 8.9	55.7 ± 8.0	55.5 ± 10.7	0.002*
Mean BMI (kg/m²)	25.0 ± 5.0	25.5 ± 4.7	25.7 ± 4.2	0.15
Education				
Low	36 (42.9)	36 (27.3)	40 (40)	0.059
Middle	38 (45.2)	72 (54.5)	40 (40)	
High	10 (11.9)	24 (18.2)	20 (40)	
No information	2	3	2	
Monthly sun bed use	17 (19.8)	36 (26.9)	28 (27.5)	0.41
No information	0	1	0	

*Significant *p*-value

^a^ *p* = 0.0007 for smokers compared with ex-smokers; *p* = 0.006 for smokers compared with never-smokers; *p* = 0.83 for ex-smokers compared with never-smokers

The smoking behavior of the patients in the current sample was compared with that in the Dutch population for which data relating to gender were available. The differences between the observed and expected smoking behaviors were small and not significant (*p* = 0.66) (Table 2).

**Table 2:** Comparison between observed smoking behavior and expected smoking behavior based on the sex distribution in the sample

		Females *n* (%)	Males *n* (%)	Total
Smokers	O	74 (86.0)	12 (14.0)	86
	E	71.2 (82.4)	15.2 (17.6)	86.4
Ex-smokers	O	106 (78.5)	29 (21.5)	135
	E	99.2 (77.6)	28.6 (22.4)	127.8
Nonsmokers	O	89 (87.3)	13 (12.7)	102
	E	98.5 (90.5)	10.3 (9.5)	108.8
*p* Value	(a)	0.50	0.48	0.66
	(b)	0.34	0.23	0.66

*O* observed number of subjects; *E* expected number of subjects; *(a)* *p* value for the comparison among the three groups; *(b)* *p* value for the comparison of never-smokers with smokers and ex-smokers combined

## Discussion

This retrospective cohort study aimed to observe whether smoking is related to cosmetic surgery. The results of the study showed that the distribution of smokers and ex-smokers in the blepharoplasty group equaled that in the Dutch population. However, the smokers were significantly younger than the ex- and never-smokers at the time of surgery. This observation appears related to the deleterious effects of cigarette smoke on facial skin. Such effects were not seen in ex-smokers. Because this study was performed at a time when all patients with dermatochalasis were reimbursed for surgical procedures, a selection bias due socioeconomic status was less likely.

This is the first study to describe an earlier need for upper-eyelid correction in smokers. It is known that smoking, age, and sun exposure are independent risk factors that contribute to wrinkle formation [9]. Sun exposure is one of the most studied factors leading to premature aging. However, cigarette smoke predisposes skin to aging more than sun exposure, and the daily use of 20 cigarettes is equivalent to almost 10 years of aging [1]. This was confirmed in a study of monozygotic twins that showed a significant cumulative increase in apparent age relative to the years of smoking [10].

Besides the effect of smoking on the skin, factors contributing to wrinkle formation may be squinting of the eyes and pursing of the lips during the inhalation of cigarette smoke [11]. Findings have shown that furrow depth is greater in smokers with 35 pack-years, but the number of furrows is fewer than with never-smokers [12]. Dermatochalasis could be the result of the large furrow depth and the decreased number of furrows around the eyes.

The molecular mechanism of aging seems to be accelerated by smoking. Smoking results in blockage of the cellular responses of the fibroblasts, leading to decreased collagen synthesis, an induced matrix metalloproteinases expression, and abnormal accumulation of elastic fibers and proteoglycans [12].

It is not known whether cessation of smoking results in partial restoration of the skin. In our study, we observed no difference between ex-smokers and never-smokers. The average time between cessation of smoking and surgery, however, was 20.4 years. We conclude that during this period, the effect of smoking on skin aging with respect to dermatochalasis had disappeared, probably due to the regenerative capacity of the skin. This is in line with other studies, which have shown that nicotine abstention results in restoration of the inflammatory cell oxidative burst [13].

## Conclusion

Although this study was limited because of its retrospective character, it strongly suggests a relation between smoking and the age at which cosmetic surgery is performed.
